# Supplementary material for: Floral Elegance Meets Medicinal Marvels: Traditional Uses, Phytochemistry, and Pharmacology of the Genus Lagerstroemia L
Source: Plants (Basel). 2024 Oct 28;13(21):3016. doi: 10.3390/plants13213016 (PMC11548200; doi:10.3390/plants13213016)
Supplement: Supplementary file 1 [file plants-13-03016-s001.zip › plants-3250362-supplementary.pdf]

Table S1. The 364 compounds have been identified from the plants of *Lagerstroemia* genus

no.	compounds	CAS	PubChem CID	species	plant parts	in vitro functions	in vivo functions	Clinical trials	ref.
**terpenoid**									
1	18-(21-ethyl-26,26-dimethylhexyl)-12-hydroxy-10-methyl-1,2,4,5,6,7,8,9,10,13,14,15,16,17-tetradecahydrochrysen-3-one			*Lagerstroemia speciosa* L.	fruit	NA	NA	NA	[1]
2	21-hydroxylupa-1,12-dien-3-one			*Lagerstroemia indica* L.	leaf	NA	NA	NA	[2]
3	24-methylenecycloartanol	1449-09-8	94204	*L. indica*	flower and leaf	antivirus, antidiabetic	antidiabetic, anti-inflammatory	NA	[3,4]
				Lagerstroemia loudonii Teijsm. & Binn	leaf				[3]
4	24-methylenecycloartanol acetate	1259-94-5	13151740	*L. speciosa*	leaf	NA	NA	NA	[5]
5	29-(36-ethyl-30-hydroxy-31-vinyloctan-29-yl)-12-methoxy-3-[(7′-methoxy-1′-oxo-2′(2″,3″,5″trihydroxyp			*L. speciosa*	fruit	NA	NA	NA	[1]

	henyl)chroman-3′-yl)oxy]-4,4,9,10,19-pentamethyl-1,2,3,4,5,6,7,8,9,10,11,14,15,16,17,18,19,20,21,22icosahydropicene-23-carbaldehyde								
6	2-hydroxy-3-oxo-24-noroleana-1,4,12-trien-28-oic acid			*L. speciosa*	fruit	anti α-glucosidase	NA	NA	[6]
7	2-hydroxy-3-oxo-24-norursa-1,4,12-triene-28-oic acid			*L. speciosa*	fruit	NA	NA	NA	[6]
8	up-20(29)-ene-1β,2α,3β-triol			*L. indica*	leaf	NA	NA	NA	[2]
9	β-carotene	7235-40-7	5280489	*L. indica*	leaf	NA	NA	NA	[7]
**sesquiterpenoid**									
10	dihydro-β-cyclopyrethrosin			*Lagerstroemia calyculata* Kurz	branch, stem bark	NA	NA	NA	[8]
11	loliolide	5989-02-6	100332	*L. indica*	stem and leaf	anti-inflammatory, anti-melanogenic, antioxidant, anti-apoptosis, anti-scratching, antitumor, lipid lowering, antidiabetic, neuroprotective activity	antioxidant	NA	[9]

12	vomifoliol	23526-45-6	5280462	*L. indica*	stem	immunosuppressant, neuroprotective, antibacterial	NA	NA	[10]
**diterpenoid**									
13	lagerstronolide		101445553	*Lagerstroemia lancasteri*	leaf and twig	NA	NA	NA	[11]
14	phytol	150-86-7	5280435	*L. indica*<br>*L. loudonii*<br>*L. speciosa*<br>*Lagerstroemia villosa* Wall. ex Kurz	leaf<br>leaf<br>leaf<br>leaf	antimicrobial, antitumor, antimutagenic, anti-teratogenic, antibiotic-chemotherapeutic, antidiabetic, antioxidant, anti-inflammatory	antitumor, immune adjuvant, antidiabetic, lipid lowering, antispasmodic, anticonvulsant, antinociceptive, anxiolytic, antidepressant, hair growth facilitator, hair fall defense, antidandruff, anti-inflammatory	NA	[3]<br>[3]<br>[12]<br>[3]
15	tinotufolins C			*L. speciosa*	leaf	NA	NA	NA	[5]
16	tinotufolins D			*L. speciosa*	leaf	NA	NA	NA	[5]
**triterpenoid**									
17	2α,3α,24-trihydroxyolean-18-ene-28-oic acid			*L. speciosa*	fruit	NA	NA	NA	[6]
18	2α,3β,24-trihydroxylup-20(29)-ene-28-oic acid			*L. speciosa*	fruit	NA	NA	NA	[6]
19	2β,3α-urs-12-en-28-oic acid			*Lagerstroemia floribunda* Jack	branch	NA	NA	NA	[8]

20	3α-acetoxy-27-hydroxylup-20(29)-en-28-oic acid methyl ester			*L. speciosa*	fruit	NA	NA	NA	[6]
21	3α-acetoxy-27-hydroxyolean-12-en-28-oic acid methyl ester			*L. speciosa*	fruit	NA	NA	NA	[6]
22	3β,23-dihydroxy-1-oxo-olean-12-en-28-oic acid			*L. indica*<br>*L. speciosa*	stem<br>leaf	NA<br>NA	NA<br>NA	NA<br>NA	[10]<br>[13]
23	3β,29-dihydroxy-olean-12-en-28-oic acid			*L. floribunda*	branch	NA	NA	NA	[8]
24	3β-hydroxy-1-oxo-olean-12-en-28-oic acid			*L. speciosa*	fruit	NA	NA	NA	[6]
25	8,26-cyclo-urs-21-en-3β,20β-diol			*L. speciosa*	fruit	NA	NA	NA	[6]
26	β-sitosterol glucoside	474-58-8	5742590	*L. speciosa*	leaf	NA	NA	NA	[13]
27	germanicyl acetate	10483-91-7	44144955	*L. lancasteri*	leaf and twig	NA	NA	NA	[14]
28	glut-5-en-3β-ol	545-24-4	9932254	*L. guilinensis*	stem	NA	NA	NA	[15]
29	jacoumaric acid		11700083	*L. lancasteri*	leaf and twig	anti leishmaniasis	NA	NA	[14]
30	lup-20(29)-en-3β,30-diol			*L. speciosa*	fruit	NA	NA	NA	[6]
31	psiguanin A			*L. speciosa*	fruit	NA	NA	NA	[6]
32	punicaone			*L. speciosa*	fruit	NA	NA	NA	[6]
33	soyasapogenol B	595-15-3	115012	*L. speciosa*	fruit	immunohemolysis, antitumor, antivirus	hepatoprotective, memory Disorders, alcoholism, anti-	NA	[6]

							obesity, anti-inflammatory		
34	squalene	111-02-4	638072	*L. loudonii*	leaf	antioxidant,	periodontal disease,	influenza	[3]
				L. speciosa	seed and leaf	antivirus, anti-inflammatory,	cholesterol-lowering, anticancer,	vaccine, skin disease,	[3]
				L. villosa	leaf	antitumor	antioxidant, antivirus, inflammatory	antidiabetic, alopecia, hyper lipemic	[3]
35	stigmasterol	83-48-7	5280794	*L. indica*	leaf	antimicrobial,	anti-inflammatory,	NA	[3]
				L. loudonii	leaf	anti-inflammatory,	antitumor, anti-diabetic,		[3]
				L. speciosa	leaf				[3]
				Lagerstroemia fauriei Koehne	leaf	antioxidant, anti α-amylase and α-glucosidase,	immunomodulatory, antiparasitic, neuroprotective		[16]
				L. villosa	leaf	anticancer			[3]
36	taraxasterol acetate	6426-43-3	13889352	*L. speciosa*	leaf	anti-inflammatory, antimalarial, antitumor, antibacterial	anti-inflammatory, analgesic, antimalarial, hepatitis	NA	[17]
**tetracyclic triterpenoid**									
37	(22E,24R)-ergosta-7,9(11),22-triene-3β,5β,6α-triol	71097-07-9	131751260	*L. speciosa*	fruit	NA	NA	NA	[6]
38	(22E,24R)-ergosta-8(14),22-diene-3β,5α,6β,7α-tetraol			*L. speciosa*	fruit	NA	NA	NA	[6]
39	(24S)-stigmast-5-ene-7β-ethoxy-3β-ol			*L. speciosa*	fruit	NA	NA	NA	[6]

40	5α,6α-epoxy-3β-hydroxy-(22E,24R)-ergosta-22-ene-7-one			*L. speciosa*	fruit	NA	NA	NA	[6]
41	5α,6α-epoxy-3β-hydrox-y-(22E,24R)-ergosta-9(11),22-diene-7-one			*L. speciosa*	fruit	NA	NA	NA	[6]
42	5α,8α-epidioxy-(22E,24R)-ergosta-6,22-diene-3β-ol			*L. speciosa*	fruit	NA	NA	NA	[6]
43	6β-methoxy-(22E,24R)-ergosta-7,22-diene-3β,5α-diol			*L. speciosa*	fruit	NA	NA	NA	[6]
44	6β-methoxy-(22E,24R)-ergosta-7,9(11),22-triene-3β,5α-diol			*L. speciosa*	fruit	NA	NA	NA	[6]
45	7-oxositosterol	2034-74-4	160608	*L. speciosa*	fruit	kidney diseases, antitumor	NA	NA	[6]
46	7α-hydroxysito-sterol			*L. speciosa*	fruit	NA	NA	NA	[6]
47	7α-methoxy-5α,6α-epoxy-(22E,24R)-ergosta-8(14),22-diene-3β-ol			*L. speciosa*	fruit	NA	NA	NA	[6]
48	7α-methoxy-stigmast-5-ene-3β-ol			*L. speciosa*	fruit	NA	NA	NA	[6]
49	7β-hydroxysitosterol	34427-61-7	161816	*L. speciosa*	fruit	kidney diseases, antitumor	NA	NA	[6]
50	7β-methoxy-stigmast-5-ene-3β-ol			*L. speciosa*	fruit	NA	NA	NA	[6]
51	daucosterol	474-58-8	5742590	*L. speciosa*	fruit and leaf		cardio protection, anti-inflammatory, liver	NA	[13]

				Lagerstroemia balansae Koehne	stem	antitumor, anti α-amylase, antioxidant	protection, antidiabetic, hypolipidemic, antitumor, neuroprotection		[18]
52	lagerenol			*L. lancasteri*	leaf and twig	NA	NA	NA	[14]
53	lagerenyl acetate			*L. lancasteri*	leaf and twig	NA	NA	NA	[14]
54	stigmast-4-ene-6β-ol-3-one			*L. speciosa*	fruit	NA	NA	NA	[6]
55	stigmastane-3β,6α-diol			*L. speciosa*	fruit	NA	NA	NA	[6]
56	γ-sitosterol	83-47-6	457801	*L. indica*	leaf	anticancer	antidiabetic	NA	[3]
				L. loudonii	leaf				[3]
				L. speciosa	seed				[19]
				L. villosa	leaf				[3]
**pentacyclic triterpenoid**									
57	23-hydroxyursolic acid	94414-19-4	14136881	*L. speciosa*	leaf	osteolytic bone disorders, renal protection, antitumor, antitubercular, anti-inflammatory	osteolytic bone disorders, anti-obesity, atherosclerosis, anti-inflammatory, anti-nociceptive effects	NA	[20]
58	7-oxo-3β-hydroxy-5,20(29)diene-24-norlupane			*L. indica*	leaf	NA	NA	NA	[2]
59	α-amyrin	638-95-9	73170	*L. speciosa*	fruit	anti-inflammatory, antioxidant, ant plasmodial	antitumor, antioxidant, antidiabetic, ant plasmodial	NA	[6]
60	alphitolic acid		12305768	*L. floribunda*	branch		anti-inflammatory	NA	[8]

		19533-92-7		*L. speciosa*	leaf	antimicrobial, antiproliferative, anti-inflammatory, anticancer, antioxidant			[21]
61	arjunic acid	31298-06-3	15385516	*L. speciosa*	fruit	antitumor, antidiabetic, antioxidant, antibacterial	antidiabetic, antihypertensive, liver protection	NA	[6]
62	arjunolic acid	465-00-9	73641	*Lagerstroemia guilinensis* S.K.Lee & L.F.Lau	stem	antibacterial, antitumor, cardio protection, antioxidant, antimalarial, treat nonalcoholic fatty liver disease	anti-depressant, treat snake bites, antiallergic and anti-asthmatic, antitumor, protect cisplatin-induced testicular injury, antimalarial, treat nonalcoholic fatty liver disease, antidiabetic	NA	[15]
				L. indica	stem				[10]
				L. speciosa	leaf				[20]
				Lagerstroemia tomentosa C.Presl	branch				[8]
63	asiatic acid	464-92-6	119034	*L. floribunda*	-	antibacterial, anti-inflammatory, antiacne, antitumor, antioxidant	antitumor, antidiabetic, antihypertensive, neuroprotective, cardioprotective, heal acute wounds	antiacne, diabetic neuropathy	[22]
				L. balansae	-				[22]
				L. calyculata	-				[22]
				Lagerstroemia cochinchinensis Pierre ex Laness.	-				[22]
				Lagerstroemia crispa Pierre ex Gagnep.	-				[22]
				L. floribunda	-				[22]

				Lagerstroemia limii Merr.	-				[22]
				L. loudonii	-				[22]
				Lagerstroemia ovalifolia Teijsm. & Binn	-				[22]
				Lagerstroemia venusta Wall	leaf				[22]
				L. speciosa	leaf				[22]
64	betulin	473-98-3	72326	*L. guilinensis*<br>*L. speciosa*	stem<br>fruit	anti-inflammatory, antitumor, leukemia, antibacterial, antivirus	analgesic, antitumor, inflammatory, antidiabetic, improve lung and liver injuries	skin care and skin disease	[15]<br>[6]
65	betulinic acid	472-15-1	64971	*L. indica*<br>*L. calyculata*<br>*L. calyculata*<br>*L. speciosa*	flower<br>branch<br>stem bark<br>fruit	antibacterial, anticancer, antivirus	antitumor, anti-inflammatory, treat leukemia, antidiabetic	skin melanoma, dysplastic naevus syndrome	[10]<br>[8]<br>[23]<br>[6]
66	corosolic acid	4547-24-4	6918774	*L. calyculata*<br>*L. crispa*<br>*L. floribunda*<br>*L. indica*<br>*L. lancasteri*<br>*L. limii*<br>*L. loudonii*<br>*L. ovalifolia*<br>*L. speciosa*	-<br>-<br>-<br>leaf<br>leaf and twig<br>-<br>fruit<br>-<br>leaf	anti leishmaniasis, antitumor, antibacterial, anti α-glucosidase,	antidiabetic, antitumor, anti-inflammatory, anti-obesity, cardiovascular, liver protection	metabolic syndrome, antidiabetic	[22]<br>[22]<br>[22]<br>[24]<br>[14]<br>[22]<br>[25]<br>[22]<br>[22]
67	friedelin	559-74-0	91472	*L. guilinensis*	stem			NA	[15]

				L. lancasteri	leaf and twig	antioxidant, anti-inflammatory, antibacterial, antitumor, antifungal	neuroprotection, cardiovascular, antitumor, antidiabetic, gastroprotective, anti-inflammatory, analgesic, antipyretic, antinociceptive, antiulcerogenic		[14]
68	hederagenin	465-99-6	73299	*L. indica*	stem	antioxidant, antivirus, anti-apoptotic, anti*α*-amylase and *α*-glucosidase	anti-inflammatory, antitumor, liver protection, hemolysis, antiatherosclerosis, diabetic nephropathy	NA	[10]
69	lageflorin			*Lagerstroemia parviflora* Roxb.	whole palnt	NA	NA	NA	[26]
70	lupeol	545-47-1	259846	*L. guilinensis*<br>*L. loudonii*<br>*L. speciosa*	stem<br>leaf<br>fruit	skin wound healing, antitumor	antitumor, anti-inflammatory, antidiabetic, inhibits skin cancer, anti-leukemic	skin infections (acne)	[15]<br>[3]<br>[6]
71	maslinic acid	4373-41-5	73659	*L. indica*<br>*L. speciosa*	stem<br>leaf	antioxidant, antimicrobial, anti-obesity	antitumor, anti-inflammatory, diabetic nephropathy	metabolic syndrome, alleviate mild knee joint pain	[10]<br>[20]
72	olean-12-en-3-one	638-97-1	6454747	*L. indica*	leaf	antibacterial, antioxidant	NA	NA	[3]
73	oleanolic acid	508-02-1	10494	*L. guilinensis*<br>*L. indica*<br>*L. loudonii*	stem<br>flower<br>fruit	antiviral, anticancer, leukemia	antitumor, liver protection, anti-inflammatory,	antidiabetic, metabolic syndrome	[15]<br>[10]<br>[27]

				L. speciosa	leaf		hypoglycemic, antidiabetic, anti-obesity, cardiovascular		[22]
74	ursolic acid	77-52-1	64945	*L. floribunda*	branch	anti-inflammatory, anti-cancer, hepato-protective, antileukemic, inhibit human lymphoma Daudi cells	vaginal contraceptive, liver disorders, antitumor, antimutagenic, antidiabetic	metabolic syndrome, primary sclerosing cholangitis, prostate cancer	[8]
				L. loudonii	fruit				[27]
				L. speciosa	leaf				[21]
75	virgatic acid	14356-51-5	14489125	*L. calyculata*	-	NA	NA	NA	[22]
				L. ovalifolia	-				[22]
				L. speciosa	leaf				[13]
**steroid**									
76	3,6-dihydroxysterol			*L. lancasteri*	leaf and twig	NA	NA	NA	[11]
77	campesterol	474-62-4	173183	*L. indica*	leaf	antitumor	cholesterol lowering, anticarcinogenic, antiangiogenic	NA	[3]
				L. loudonii	leaf				[3]
				L. speciosa	leaf				[3]
				L. villosa	leaf				[3]
78	cycloartenol	469-38-5	92110	*L. indica*	leaf	antitumor	anti-inflammatory, antidiabetic	NA	[3]
79	sitosterol	83-46-5	222284	*L. indica*	root	antioxidant, cardioprotective	anti-inflammatory, antitumor, hepatoprotective, antidiabetic, skin disease	hypercholesterolemia, gingival recession, benign prostatic hyperplasia	[28]
				L. lancasteri	leaf and twig				[14]
				L. speciosa	leaf and fruit				[1,5]
				L. balansae	stem				[18]
				L. calyculata	stem bark				[23]
				L. floribunda	branch				[8]

				L. guilinensis	stem				[15]
				L. fauriei	leaf				[16]
80	stigmast-3,6-dione	22149-69-5	13992092	*L. speciosa*	fruit	antibacterial	NA	NA	[6]
81	β-sitosterol acetate		521199	*L. speciosa*	leaf	NA	NA	NA	[17]
**phenolic**									
82	2-Methoxy-4-vinylphenol	7786-61-0	332	*L. speciosa*	leaf	anti-inflammatory, antioxidant, antimicrobial	insect repellent	NA	[3]
83	4-Amino-benzoic acid	150-13-0	978	*L. indica*	leaf	NA	NA	NA	[7]
84	benzoic acid	65-85-0	243	*L. indica*	leaf	antibacterial	NA	NA	[7]
85	caffeic acid ethyl ester	102-37-4	5317238	*L. speciosa*	leaf	anti-inflammatory, antitumor	anti-inflammatory, antitumor, anti-inflammatory	NA	[29]
86	catechin	154-23-4	9064	*L. indica*	leaf	anticancer, anti-adipogenic, anti-inflammatory, hypolipidemic, antivirus, antibacterial	antidiabetic, antitumor, neurodegenerative diseases, analgesic, liver protection, alcoholism	anti-obesity, prostate cancer, influenza infection	[7]
87	catechol	120-80-9	289	*L. indica*	leaf	antioxidant, anti-inflammatory, antibacterial	hemostatic, antitumor	NA	[7]
88	epicatechin	490-46-0	72276	*L. indica*	root and leaf	anti-inflammatory, antitumor	neuroprotective, antidiabetic, antitumor, anti-inflammatory, anti-angiogenesis, apoptosis,	antidiabetic, becker muscular dystrophy, chronic kidney disease, hypertension,	[30]

							hepatoprotective, anti-obesity	heart failure, vasodilation	
89	isotachioside	31427-08-4	15098566	*L. indica*	stem	NA	NA	NA	[10]
90	methyl gallate	99-24-1	7428	*L. indica*	flower	antioxidant, antibacterial, antitumor, anti-inflammatory	antitumor, neuroprotective, hepatoprotective, cardioprotective, anti-inflammatory, nephroprotective	NA	[31]
91	protocatechuic acid	99-50-3	72	*L. indica*	root and stem	cardioprotective, antioxidant, anti-inflammatory, neuroprotective, antibacterial, antiviral	antitumor, metabolic syndrome, ant osteoporotic, analgesia, antiaging, preservation of liver, kidneys, and reproductive functions	immunity, prosthetic joint infection, renal disease, prosthesis-related infections	[30]
92	tachioside	109194-60-7	11962143	*L. indica*	stem	NA	NA	NA	[10]
93	vanillin	121-33-5	1183	*L. floribunda*<br>*L. speciosa*<br>*L. indica*	flower and fruit<br>flower<br>stem	neuroprotective, antioxidant, antibacterial	neuroprotective, antitumor, anti-inflammatory, alcoholism, treat major depressive disorder, antidiabetic, antinociceptive	mild-to-moderate COVID-19, neonatal hypoxic conditions, trachoma	[32]<br>[32]<br>[10]
**phenolic acid**									
94	(R)-lasiodiplodin		11833217	*L. speciosa*	fruit	antibacterial, anti-inflammatory	antibacterial, anti-inflammatory	NA	[6]

95	3,4-dihydroxybenzaldehyde	139-85-5	8768	*L. speciosa*	fruit	antibacterial, anti-inflammatory, treat cardiomyocyte senescence, antitumor	heal oral ulcers, treat obstructive nephropathy, prevent ischemic injury, antitumor, anti-fibrosis, diabetes complications	NA	[6]
96	4-hydroxybenzoic acid	99-96-7	135	*L. speciosa*<br>*L. indica*	leaf and fruit<br>root	antitumor, anti-inflammatory, neurodegeneration, anti-glycogen phosphorylase α, antibacterial, antifungal	antidiabetic, dermatitis	safe excipient exposure in neonates and small children	[33]<br>[30]
97	4-hydroxymellein	33788-22-6	169539	*L. speciosa*	fruit	antibacterial, antileukemia	NA	NA	[6]
98	5-heptylresorcinol	500-67-4	68142	*L. speciosa*	fruit	NA	NA	NA	[6]
99	cinnamic acid	140-10-3	444539	*L. indica*	leaf	anti-microbial, anti-oxidative, anti-inflammatory	anti-tumor, neuroprotective, anti-diabetic, anti-parasitic, anti-inflammatory, antidiabetic, anti-obesity, cardioprotective, antimalarial	antidiabetic	[7]
100	ethyl gallate	831-61-8	13250	*L. speciosa*	leaf and fruit	antivirus, antitumor, ant plasmodial, anticariogenic, antioxidant	anti-inflammatory, antitumor, acute lung injury	NA	[6]

101	ferulic acid	537-98-4	445858	*L. indica*	leaf	anti-inflammatory, antioxidant	anti-inflammatory, antitumor, neuroprotective, antidiabetic, anti-obesity, cardiovascular diseases, anti-depressive, liver protection	behavioral and psychiatric symptoms of dementia	[7]
102	*p*-hydroxybenzaldehyde	123-08-0	126	*L. speciosa*	fruit	NA	NA	NA	[6]
103	vanillic acid	121-34-6	8468	*L. indica*	leaf and stem	antioxidant, antibacterial	anti-inflammatory, antidiabetic, antitumor, neurodegenerative, cardiovascular diseases, antiparasitic, anti-obesity, nephroprotective, antinociceptive, anti-asthma	NA	[7]
**polyphenol**									
104	1,2,3-Benzenetriol	87-66-1	1057	*L. loudonii*<br>*L. speciosa*<br>*L. indica*<br>*L. fauriei*	leaf<br>leaf<br>root<br>leaf	antitumor, anti-inflammatory, antibacterial, antioxidant	antitumor, anti-inflammatory, anti-obesity, antimutagenic, hepatoprotective	NA	[3]<br>[3]<br>[30]<br>[16]
105	3,3'-di-O-methylellagic acid	2239-88-5	5488919	*L. speciosa*	leaf	anticancer, antibacterial, antioxidant		NA	[33]
106	3,4,3'-tri-O-methyl flavellagic acid	13756-49-5	14412544	*L. speciosa*	leaf	NA	NA	NA	[21]

107	3,4,8,9,10-pentahydroxydibenzo[b,d]pyran-6-one	91485-02-8	18504424	*L. speciosa*	leaf	antibacterial	NA	NA	[33]
108	3-O-methylellagic acid	51768-38-8	13915428	*L. fauriei*<br>*L. speciosa*	leaf<br>leaf	antifungal	NA	NA	[16]<br>[33]
109	ellagic acid	476-66-4	5281855	*L. fauriei*<br>*L. floribunda*<br>*L. indica*<br>*L. ovalifolia*<br>*L. speciosa*<br>*L. fauriei*	leaf<br>flower<br>flower<br>-<br>leaf<br>leaf	antioxidant, anti-proliferative, antibacterial, antitumor, antivirus	antitumor, neuroprotective, anti-inflammatory, antidepressant, antidiabetic, anti-thrombosis, metabolic syndrome, liver protection	gut microbiome, metabolic syndrome, colon-rectal cancer, HPV Infection	[34]<br>[32]<br>[34]<br>[22]<br>[32]<br>[34]
110	gallic acid	149-91-7	370	*L. balansae*<br>*L. floribunda*<br>*L. indica*<br><br>*L. speciosa*	stem<br>flower<br>flower, leaf, stem<br>leaf	antibacterial, antitumor, anti-inflammatory, neuroprotective, anticariogenic	antitumor, inflammatory, antidiabetic, anti-obesity, antidepressant, liver protection	NA	[35]<br>[32]<br>[7,10,31]<br><br>[33]
111	lutein	127-40-2	5281243	*L. speciosa*	leaf	anti-inflammatory, eye disease	antitumor, anti-obesity, eye disease, neuroprotective	eye fatigue, macula lutea opacity, macular pigment, multiple sclerosis, carotid atherosclerosis	[5]
112	valoneaic acid dilactone	60202-70-2	10151874	*L. speciosa*	leaf	anti α-amylase, inhibit xanthine-oxidase	NA	NA	[36]

flavonoid

113	4',7-dihydroxyflavone	2196-14-7	5282073	*L. indica*	stem and leaf	anti-asthma	anti leishmania	NA	[9]
114	acacetin	480-44-4	5280442	*L. indica*	leaf	antitumor, anti-inflammatory, antibacterial	antitumor, anti-inflammatory, improve lung injury, arthritis, anti-obesity, hepatic protection	NA	[7]
115	apigenin	520-36-5	5280443	*L. indica*	leaf	antitumor, antioxidant, anti-inflammatory, antibacterial, anti-leukemia., anti α-glucosidase	antitumor, neurodegenerative, improve atherosclerosis, anti-obesity, antidiabetic	sepsis, health benefits in high risk breast clinic patients, cardiovascular	[7]
116	epicatechin gallate	1257-08-5	107905	*L. floribunda*<br>*L. speciosa*	flower<br>flower	antitumor, atherosclerosis, antibacterial, antioxidant, anti α-glucosidase	antitumor, atherosclerosis	NA	[32]<br>[32]
117	epigallocatechin	970-74-1	72277	*L. speciosa*	bark	antioxidant, anti-inflammatory, anti α-glucosidase and α-amylase, nerve degeneration	antitumor, nerve degeneration, anti-inflammatory	NA	[37]
118	hyperin	482-36-0	5281643	*L. speciosa*<br>*L. ovalifolia*	leaf<br>-	anti-inflammatory, antioxidant	antitumor, anti-inflammatory, diabetes complications, neurodegenerative diseases, anti-obesity	NA	[17]<br>[22]
119	iso-orientin	4261-42-1	114776	*L. indica*	leaf	antioxidant, antibacterial,	anti-inflammatory, antitumor,	NA	[38]

						antidiabetic, neurodegenerative diseases	neurodegenerative diseases, anti-obesity, anti-asthma, liver protection		
120	isovitexin	38953-85-4	162350	*L. indica*	leaf	antioxidant, anticancer	antitumor, anti-inflammatory, anti α-glucosidase	NA	[38]
121	kaempferol	520-18-3	5280863	*L. indica*<br>*L. speciosa*	leaf<br>leaf	antioxidant, anticancer, anti-inflammatory	anti-inflammatory, antitumor, cardioprotective, anti-Alzheimer	female sexual dysfunction	[7]<br>[33]
122	kaempferol-3-O-α-L-rhamnoside	482-39-3	5316673	*L. ovalifolia*	-	antioxidant, anti-inflammatory	NA	NA	[22]
123	lageracetal	5921-80-2	22210	*L. indica*<br>*L. speciosa*<br>*L. fauriei*	leaf<br>leaf<br>leaf	NA	NA	NA	[7]<br>[34]<br>[34]
124	luteolin	491-70-3	5280445	*L. indica*	leaf	antioxidant, anti-inflammatory	anti-inflammatory, antitumor, nephroprotective, angiogenesis inhibitor, apoptosis inducer, antidiabetic, neurodegenerative diseases	tongue neoplasms, schizoaffective disorder, memory, metabolic syndrome, frontotemporal dementia, autism spectrum disorders	[38]
125	luteolin-7-O-$^4C_1$-β-D-glucoside	5373-11-5	5280637	*L. indica*	leaf	anticancer, anti-inflammatory	antidiabetic, atherosclerosis, lipid-lowering, liver protection, antitumor	NA	[38]

126	morusin	62596-29-6	5281671	*L. indica*	stem and leaf	antitumor, anti-inflammatory, antibacterial, antioxidant	antitumor, anti-inflammatory	NA	[9]
127	morusinol	62949-93-3	5481968	*L. indica*	stem and leaf	antitumor	cardiovascular disease	NA	[39]
128	naringenin	480-41-1	439246	*L. indica*	leaf	anti-inflammatory, antioxidant, anti-infective, antibacterial	antitumor, hepatoprotective, neuroprotective, metabolic disorders, atherosclerosis, anti-obesity, cardiovascular diseases	HCV Infection, bone fractures, energy expenditure,	[7]
129	neocyclomorusin	62596-35-4	5481973	*L. indica*	stem and leaf	NA	NA	NA	[39]
130	nudiposide	62058-46-2	14521040	*L. indica*	leaf	neuroprotective	NA	NA	[7]
131	orientin	28608-75-5	5281675	*L. indica*	leaf	antioxidant, antiaging, antiviral, antibacterial, anti-inflammation, diabetic nephropathy, antitumor, antithrombotic	vasodilatation and cardioprotective, radiation protective, neuroprotective, antidepressant-like, anti-adipogenesis, antinociceptive, antitumor, antithrombotic	COVID-19	[38]
132	quercetin	117-39-5	5280343	*L. floribunda*<br>*L. indica*<br>*L. speciosa*	flower<br>leaf<br>leaf	anti-inflammatory, antitumor, antibacterial	anti-inflammatory, neuroprotective, antitumor, cardiovascular diseases,	chronic obstructive pulmonary disease, problem of aging,	[32]<br>[7]<br>[33]

							atherosclerosis, diabetic	gastroesophageal reflux disease, cardiometabolic syndrome, schizophrenia	
133	quercetin-3-O-β-D-glucoside	21637-25-2	5484006	*L. ovalifolia*<br>*L. speciosa*	-<br>leaf	NA	NA	NA	[22]<br>[33]
134	quercetin-7-glucoside	491-50-9	5282160	*L. speciosa*	leaf	antivirus	NA	NA	[40]
135	quercetrin	522-12-3	5280459	*L. indica*	leaf	antioxidant, antitumor	anti-inflammatory, antidiabetic, antitumor	Alzheimer disease	[7]
136	rhamnetin	90-19-7	5281691	*L. indica*	leaf	antioxidant, anti-inflammatory, antibacterial, anti-melanogenic	anti-inflammatory, anticancer, albinism, cardioprotective	NA	[7]
137	rutin	153-18-4	5280805	*L. indica*	leaf	antioxidant, anti-inflammatory, anti-fibrotic, antivirus	anti-inflammatory, antitumor, antidiabetic, neurodegenerative diseases, venous insufficiency	end stage renal disease, type 2 diabetes mellitus, colorectal cancer	[7]
138	vitexin	3681-93-4	5280441	*L. indica*	leaf	anticancer, anti-inflammatory, alleviate heat stress	anti-inflammatory, antitumor, alleviate non-alcoholic fatty liver disease, anti-Alzheimer, neuroprotective, ischemia, liver protection, inhibit pain	obesity	[38]
**tannin**									

139	2,3-(S)-hexahydroxydiphenoyl-α/β-D-glucose	83541-37-1	14035453	*L. speciosa*	leaf	NA	NA	NA	[33]
140	3-O-caffeoylquinic acid	327-97-9	1794427	*L. speciosa*<br>*L. indica*	leaf<br>leaf	antioxidant, anti-inflammatory,	anticancer, anti-inflammatory, anti-obesity, antidiabetic, hepatoprotective, anti-depressive-like behaviors, neurodegenerative, liver protection, metabolic syndrome	NA	[41]<br>[7]
141	3,4,3'-tri-O-methylellagic acid	1617-49-8	5281860	*L. speciosa*<br>*L. balansae*	leaf<br>stem	NA	NA	NA	[33]<br>[35]
142	4,6-(S)-HHDP-D-glucose	36378-48-0		*L. speciosa*	leaf	NA	NA	NA	[41]
143	brevifolin carboxylic acid	18490-95-4	9838995	*L. balansae*<br>*L. speciosa*	stem<br>leaf	anticancer, antibacterial, anti-parasitic	NA	NA	[35]<br>[41]
144	caffeic acid	501-16-6	689043	*L. indica*<br>*L. speciosa*	leaf<br>leaf	anti α-glucosidase, anti-inflammatory, antibacterial, antioxidant	anti-inflammatory, antitumor, antidiabetic, anti-Alzheimer disease, neurodegenerative, cardiovascular diseases, anti-obesity, atherosclerosis, Parkinson disease	esophagus cancer, stage III	[7]<br>[33]
145	castalagin	24312-00-3	168165	*L. speciosa*	leaf and fruit	protect SH-SY5Y cells from Aβ42-mediated death,	antitumor, anti-leishmania, anti-	NA	[41]

						antioxidant, antibacterial	herpetic, gastroprotective		
146	casuariin		14035442	*L. speciosa*	leaf	anti-diabetes complications, anti-cancer	NA	NA	[41]
147	casuarinin	79786-01-9	442673	*L. speciosa*	leaf	anti-herpesvirus, antitumor, antioxidant, improve sarcopenia, anti-diabetes complications	anti-inflammatory, improve sarcopenia, antitumor	NA	[33]
148	flosin A		101596476	*L. speciosa*	leaf	NA	NA	NA	[42]
149	flosin B			*L. speciosa*	leaf	NA	NA	NA	[43]
150	gemin D	84744-46-7	471119	*L. speciosa*	fruit	antioxidant, antitumor	antitumor	NA	[44]
151	grandinin	115166-32-0	492392	*L. speciosa*	fruit	inhibit lung malignancies, antitumor	antidiabetic	NA	[44]
152	hippophaenin A	133379-12-1	16130427	*L. speciosa*	fruit	NA	NA	NA	[44]
153	lagerstroemin		153274285	*L. speciosa*	leaf and fruit	antidiabetic	NA	NA	[33]
154	pedunculagin	7045-42-3	442688	*L. speciosa*	leaf	anti-inflammatory, inhibit 5α-reductase, antibacterial, anti-hemolytic, anti-skin aging, hypertension	anti-inflammatory, inhibit lipid peroxidation	NA	[41]

155	pterocarinin A		14731325	*L. speciosa*	leaf	antioxidant	NA	NA	[41]
156	reginin A			*L. speciosa*	leaf	antidiabetic	NA	NA	[43]
157	stachyurin	81739-27-7	157395	*L. speciosa*	leaf	anticancer	NA	NA	[33]
158	tannic acid	1401-55-4	16129778	*L. speciosa*	leaf	anti α-glucosidase, antioxidant, antibacterial	inhibit colitis	NA	[45]
**anthocyanin**									
159	cyanidin 3-O-glucoside	7084-24-4	197081	*L. speciosa*	petal	anti α-glucosidase, antioxidant, prevent colon cancer, anti-inflammatory, antivirus	hypotensive and vasodilatory effect, antivirus	NA	[46]
160	delphinidin-3-arabinoside	28500-01-8	137347179	*L. indica*	flower	anti enzyme dipeptidyl peptidase-IV	NA	NA	[31]
161	delphinidin-3-O-glucoside	50986-17-9	443650	*L. indica*	petal	antitumor, cardiovascular diseases	NA	NA	[47]
162	malvidin 3,5-di-O-glucoside	16727-30-3	441765	*L. speciosa*	petal	NA	NA	NA	[46]
163	malvidin 3-O-glucoside	18470-06-9	443652	*L. indica*<br>*L. speciosa*	petal<br>petal	antioxidant, anti-inflammatory	antidiabetic	NA	[47]<br>[46]
164	1,2,3,4,6-Penta-O-galloyl-β-D-glucose	14937-32-7	65238	*L. speciosa*	leaf	anti-parasitic, neurodegenerative disorders, antioxidant,	antidiabetic, hepatoprotective, antitumor, anti-inflammatory	NA	[48]

						antitumor, antivirus			
165	petunidin 3-O-glucoside	71991-88-3	443651	*L. indica*	petal	antioxidant, anti-obesity, neuroprotective	NA	NA	[47]
166	petunidin-3-arabinoside	679429-94-8	91810653	*L. indica*	flower	NA	NA	NA	[31]
**alkaloid**									
167	2-hydroxy-trans-4-3',4'-di-methoxyphenyl-cis-quinolizidine			*L. fauriei*	leaf	NA	NA	NA	[49]
168	2-hydroxy-trans-4-3',4'-di-methoxyphenyl-trans-quinolizidine			*L. fauriei*	leaf	NA	NA	NA	[49]
169	5-epi-dihydrolyfoline		42640298	*L. indica*	aerial part	NA	NA	NA	[50]
170	5-methyluridine	1463-10-1	445408	*L. speciosa*	bark	NA	NA	NA	[37]
171	cryogenine	103-03-7	61002	*L. fauriei*	leaf	inhibit prostaglandin synthetase	NA	NA	[49]
172	dihydrolyfoline		42640297	*L. indica*	aerial part	NA	NA	NA	[50]
173	lagerindicine			*L. indica*	flower	NA	NA	NA	[4]
174	lythridine	15299-77-1	137699649	*L. fauriei*	leaf	NA	NA	NA	[49]
175	lythrine	5286-10-2	6441299	*L. fauriei*	leaf	NA	hydro diuretic	NA	[49]
176	pterolactam	38072-88-7	181561	*L. indica*	flower	antibacterial	NA	NA	[4]
177	sarusubine A		23584968	*L. fauriei*	leaf	NA	NA	NA	[51]
178	subcosine-I		11762590	*L. fauriei*	leaf	NA	NA	NA	[49]
179	subcosine-II		11730540	*L. fauriei*	leaf	NA	NA	NA	[49]

**phenylpropanoid**									
180	(2S)-3,3-di-(4-hydroxy-3-methoxyphenyl)-propane-1,2-diol			*L. speciosa*	fruit	inhibit nitric oxide production	NA	NA	[52]
181	(7R,8S)-3,5′-dimethoxy-4′,7-epoxy-8,3′-neolignane-5,9,9′-triol			*L. speciosa*	fruit	inhibit nitric oxide production	NA	NA	[52]
182	1-(4-hydroxy-3-methoxyphenyl)-3-hydroxy-propan-1-one			*L. speciosa*	fruit	NA	NA	NA	[52]
183	1,2-bis-(4-hydroxy-3-methoxyphenyl)-1,3-propanediol			*L. speciosa*	fruit	NA	NA	NA	[52]
184	2,3-bis-(4-hydroxy-3-methoxyphenyl)-1,3-propanediol			*L. speciosa*	fruit	NA	NA	NA	[52]
185	2,3-bis-(4-hydroxy-3-methoxyphenyl)-3-methoxypropanol			*L. speciosa*	fruit	NA	NA	NA	[52]
186	2,3-dihydroxy-1-(4-hydroxy-3-methoxyphenyl)-propan-1-one	168293-10-5	15765124	*L. speciosa*	fruit	NA	NA	NA	[52]
187	curcasinlignan A		101571103	*L. speciosa*	fruit	NA	NA	NA	[52]
188	curcasinlignan B		101571104	*L. speciosa*	fruit	NA	NA	NA	[52]
189	evofolin B	168254-96-4	5317306	*L. speciosa*	fruit	inhibit superoxide generation and elastase release	NA	NA	[52]

190	ficusal	321991-55-3	10496641	*L. speciosa*	fruit	anti a-glucosidase, antitumor	NA	NA	[52]
191	pinoresinol	487-36-5	73399	*L. speciosa*	fruit	antifungal, anti-inflammatory, antioxidant, antitumor	analgesic	NA	[52]
192	secoisolariciresinol	29388-59-8	65373	*L. speciosa*	fruit	antitumor, antioxidant, neuroprotective	NA	breast cancer	[52]
193	syringaresinol	1177-14-6	100067	*L. speciosa*	fruit	anti acetylcholinesterase, antioxidant, anti-inflammatory	anti-inflammatory, diabetic nephropathy, diabetes complications, neuralgia	NA	[52]
194	p-coumaric acid	501-98-4	637542	*L. speciosa*<br>*L. indica*	leaf<br>root	antibacterial, antioxidant, hepatoprotective, antitumor, antifibrillatory	hepatoprotective, anti-inflammatory, anti-obesity, neurodegenerative diseases	NA	[33]<br>[30]
**coumarin**									
195	6,7-dihydroxy coumarin	305-01-1	5281416	*L. speciosa*	leaf	antioxidant, inhibit purified Human Serum Paraoxonase 1	NA	NA	[21]
196	clauslactone-K	207602-26-4	10689615	*L. calyculata*<br>*L. calyculata*	branch<br>stem bark	NA	NA	NA<br>NA	[8]<br>[23]
**lignan**									
197	(+)-dihydrodehydrodiconi			*L. speciosa*	leaf	NA	NA	NA	[21]

	feryl alcohol 9'-O-sulfate								
198	(7S,8R)-dihydrodehydrodiconiferyl alcohol	28199-69-1	384679	*L. speciosa*	leaf	NA	NA	NA	[53]
199	ent-isolariciresinol	110268-37-6	1023563	*L. floribunda*	branch	NA	NA	NA	[8]
200	gomisin	58546-56-8	151529	*L. balansae*	stem	neurodegenerative, anti-inflammatory, antitumor, antioxidant	neurodegenerative, anti-inflammatory	NA	[18]
201	lingueresinol			*L. floribunda*	branch	NA	NA	NA	[8]
202	lyoniside	34425-25-7	14521039	*L. balansae*	stem	anti leishmaniasis	anti leishmaniasis	NA	[18]
203	matairesinol	580-72-3	119205	*L. indica*	stem and leaf	neuroprotection	NA	NA	[9]
**fatty acid**									
204	14-methylhexadecanoic acid	5918-29-6	22207	*L. speciosa*	seed	NA	NA	NA	[54]
205	cis-11-Eicosenoic acid	5561-99-9	5282768	*L. speciosa*	seed	antibacterial	NA	NA	[54]
206	cis-13-Docosenoamide	112-84-5	5365371	*L. speciosa*	seed	inhibit acetylcholinesterase	NA	NA	[54]
207	cis-Vaccenic acid	506-17-2	5282761	*L. speciosa*	seed	antimalarial, antibacterial	antidepressant	healthy diet	[54]
208	coumaric acid	614-60-8	637540	*L. indica*	stem	antivirus, anti-inflammatory, antibacterial	antitumor, anti-inflammatory	colon health	[10]
209	docosanoic acid	112-85-6	8215	*L. speciosa*	seed	NA	NA	NA	[19]

210	dodecanoic acid	143-07-7	3893	*L. speciosa*	seed	antitumor, antibacterial	anti-inflammatory, antitumor, anti-obesity, neuroprotection	generalized reward sensitivity, emotion induction	[19]
211	eicosa-11,13-dienoic acid			*L. speciosa*	seed	NA	NA	NA	[54]
212	eicosanoic acid	506-30-9	10467	*L. speciosa*	seed	NA	NA	NA	[19]
213	hexadecanoic acid	57-10-3	985	*L. indica*	leaf			NA	[3]
				L. loudonii	leaf				[3]
				L. villosa	leaf				[3]
214	linoleic acid	60-33-3	5280450	*L. indica*	leaf	anti-inflammatory, antitumor, antibacterial	atherosclerosis, anti-obesity, cardiovascular disease	atherosclerosis, cardiovascular disease, multiple sclerosis, cystic fibrosis, obesity, advanced solid tumors, asthma, metabolic syndrome	[3]
				L. speciosa	seed				[19]
215	linolenic acid	463-40-1	5280934	*L. indica*	leaf	antifungal, antitumor, antibacterial	anti-inflammatory, cardiovascular diseases, anti-obesity, anti-depressive, metabolic syndrome	hypertension, cardiovascular disease, memory performance of toddlers	[3]
				L. loudonii	leaf				[3]
				L. speciosa	leaf				[3]
216	palmitic acid			*L. indica*	leaf	NA	NA	dyslipidemia	[3]
				L. loudonii	leaf				[3]
				L. speciosa	seed; leaf; bark				[19]
				L. villosa	leaf				[3]

217	oleic acid	112-80-1	445639	*L. speciosa*	seed	anti-inflammatory, antitumor	liver protection, anti-obesity, improve lung Injury, antitumor, cardiovascular diseases	nutritional intervention, inflammation	[12,55]
218	stearic acid	57-11-4	5281	*L. speciosa*	seed and bark	antitumor	anti-obesity, liver protection	dyslipidemia	[37,56]
				L. indica	leaf				[3]
219	tetracosanoic acid	557-59-5	11197	*L. speciosa*	seed	NA	NA	NA	[19]
220	tetradecanoic acid	544-63-8	11005	*L. speciosa*	seed	antibacterial, anti-inflammatory	NA	NA	[19]
221	tricosanoic acid	2433-96-7	17085	*L. speciosa*	seed	NA	NA	NA	[19]
222	α-Hydroxymyristic acid	2507-55-3	1563	*L. speciosa*	leaf	NA	NA	NA	[3]
**amino acid**									
223	Ala	56-41-7	5950	*L. speciosa*	seed	anti-HIV, anti-inflammatory	antidiabetic, anti-obesity, antitumor	nonalcoholic steatohepatitis, fructose intolerance	[57]
224	Arg	74-79-3	6322	*L. speciosa*	seed	antioxidant, anti-inflammatory, antitumor	cardiovascular diseases, atherosclerosis, anti-inflammatory, wound healing, diabetic kidney disease	heart transplant, COVID-19, anemia, sickle cell disease, rheumatoid arthritis, hypertension, metabolic syndrome, schizophrenia, erectile dysfunction, etc	[57]

225	Asp	56-84-8	5960	*L. speciosa*	seed	antitumor, anti-inflammatory	neurologic disorders, antidiabetic, hepatoprotective, , anti-inflammatory	NA	[57]
226	Cys	52-90-4	5862	*L. speciosa*	seed	antitumor, antioxidant, anti-inflammatory	hepatoprotective, cardiovascular diseases, neurodegenerative	gastritis, uremia, breast milk, oxidative injury in critically ill babies, erythropoietic protoporphyria, cluster headache	[57]
227	Glu	56-86-0	33032	*L. speciosa*	seed	neurodegenerative	neurodegenerative, anti-depressive	kidney cancer, nutrition intervention, vertigo	[57]
228	Gly	56-40-6	750	*L. speciosa*	seed	antioxidant, anti-inflammatory, antitumor	neurodegenerative, anti-depressive, antidiabetic, schizophrenia, ischemia	psychotic disorders, morbid obesity, COVID-19, cystic fibrosis, obsessive compulsive disorder, diabetes mellitus, etc	[57]
229	His	71-00-1	6274	*L. speciosa*	seed	antitumor, anti-inflammatory, antibacterial	anti-inflammatory, immunologic function, metabolic syndrome, antitumor	HARS syndrome, Alzheimer disease, Kinky Hair syndrome, rosacea	[57]
230	Ile	73-32-5	6306	*L. speciosa*	seed	promote protein synthesis,	muscle repair, antidiabetic,	acute infectious diarrhea in	[57]

						antitumor, anti-inflammatory	antidepressive, anti-inflammatory, anti-obesity	children, hyperglycemia, diabetic	
231	Leu	61-90-5	6106	*L. speciosa*	seed	antitumor, anti-inflammatory	antitumor, antidiabetic, anti-obesity, sarcopenia	major depression, Diamond Blackfan anemia, muscle protein synthesis, antidiabetic, anti-obesity, menstrual cycle	[57]
232	Lys	56-87-1	5962	*L. speciosa*	seed	antibacterial, antivirus	antivirus, antidepressive, anti-inflammatory	adrenal secretion, breastfeeding, schizophrenia, diarrhea, oral complications of chemotherapy, major depression, etc	[57]
233	Met	63-68-3	6137	*L. speciosa*	seed	antioxidant, antitumor	anti-obesity, antidepressant and anxiolytic	metabolic syndrome, breast cancer, urinary tract infection	[57]
234	Phe	63-91-2	6140	*L. speciosa*	seed	anti-inflammatory	antidepressant, antidiabetic, anti-Alzheimer	phenylketonuria	[57]
235	Pro	147-85-3	145742	*L. speciosa*	seed	wound healing, antibacterial, antivirus, antidepressant	wound healing, anti-inflammatory, neurodegenerative, antidiabetic	burns, chronic kidney disease	[57]

236	Ser	56-45-1	5951	*L. speciosa*	seed	anti-inflammatory	antitumor, neurodegenerative diseases, antidepressant, antidiabetic, Parkinson disease	amyotrophic lateral sclerosis, Alzheimer disease, schizophrenia, non-alcoholic fatty liver disease, major depressive disorder, etc	[57]
237	Thr	72-19-5	6288	*L. speciosa*	seed	antitumor	neurodegenerative diseases, anti-inflammatory, anti-obesity	ulcerative colitis, diaphragmatic hernia	[57]
238	Trp	73-22-3	6305	*L. speciosa*	seed	antitumor, anti-inflammatory	antidepressant, neurodegenerative diseases, autoimmune diseases	post-operative delirium, melatonin deficiency, brain tumors, postoperative pain, improve sleep quality	[57]
239	Tyr	60-18-4	6057	*L. speciosa*	seed	antioxidant	antidepressant, neurodegenerative diseases	stress, psychological, fibromyalgia, Restless Legs Syndrome, blood pressure, glucose tolerance	[57]

240	Val	72-18-4	6287	*L. speciosa*	seed	anemia	promote muscle recovery, regulate blood sugar	maple syrup urine disease, end stage renal disease	[57]
**acyclic hydrocarbon**									
241	Nonacosane	630-03-5	12409	*L. loudonii*	fruit	NA	NA	NA	[27]
242	Octacosane	630-02-4	12408	*L. loudonii*	leaf	antioxidant, wound excision closure	NA	NA	[3]
				L. villosa	leaf				[3]
243	Octadecane	593-45-3	11635	*L. indica*	leaf	antifungal	NA	NA	[3]
				L. loudonii	leaf				[3]
				L. speciosa	leaf and seed				[3]
				L. villosa	leaf				[3]
244	Pentacosane	629-99-2	12406	*L. speciosa*	leaf	NA	NA	NA	[3]
				L. villosa	leaf				[3]
245	Tetracosane	646-31-1	12592	*L. speciosa*	leaf	NA	NA	NA	[3]
246	Tetradecane	629-59-4	12389	*L. speciosa*	seed	NA	NA	NA	[19]
247	Tetratetracontane	7098-22-8	23494	*L. loudonii*	leaf	NA	NA	NA	[3]
248	Tetratriacontane	14167-59-0	26519	*L. indica*	leaf	NA	NA	NA	[3]
				L. loudonii	leaf				[3]
				L. villosa	leaf				[3]
249	Tricosane	638-67-5	12534	*L. speciosa*	leaf	NA	NA	NA	[3]
**alkane**									
250	Docosane	629-97-0	12405	*L. speciosa*	leaf	NA	NA	NA	[3]
251	Dodecane	112-40-3	8182	*L. speciosa*	seed	NA	NA	NA	[19]
252	Heptacosane	593-49-7	11636	*L. loudonii*	fruit	NA	NA	NA	[27]
253	Hexadecane	544-76-3	11006	*L. speciosa*	seed	NA	NA	NA	[19]
254	Pentatriacontane	630-07-9	12413	*L. guilinensis*	stem	NA	NA	NA	[15]
**miscellaneous**									
255	1-Dodecanol	112-53-8	8193	*L. speciosa*	seed	antibacterial	NA	NA	[19]

256	1-Hexadecanol	36653-82-4	2682	*L. speciosa*	seed	NA	NA	NA	[19]
257	n-amyl alcohol	71-41-0	6276	*L. fauriei*	leaf	NA	NA	NA	[34]
				L. indica	leaf				[7]
				L. speciosa	leaf				[34]
258	5-Hydroxymethylfurfural	67-47-0	237332	*L. indica*	leaf	neuron protection	NA	hypoxia, sickle cell disease	[3]
				L. loudonii	leaf				[3]
				L. speciosa	leaf and fruit				[3]
				L. villosa	leaf				[3]
259	hexadecanamide	629-54-9	69421	*L. indica*	leaf	NA	anti-inflammatory	NA	[3]
				L. loudonii	leaf				[3]
				L. speciosa	leaf				[3]
				L. villosa	leaf				[3]
260	1,4-dihydroxy-2-methoxy-7-methyl anthracene-9,10-dione	67402-63-5		*L. speciosa*	endophytic fungus	NA	NA	NA	[58]
261	naphthalene	91-20-3	931	*L. speciosa*	seed	NA	NA	NA	[19]
262	Octadecanamide	124-26-5	31292	*L. loudonii*	leaf	NA	NA	NA	[3]
				L. speciosa	leaf				[3]
				L. villosa	leaf				[3]
263	oleamide	301-02-0	5283387	*L. indica*	leaf	antitumor, anti-inflammatory	anti Alzheimer disease, antiallergic, hypnotic, anti-obesity, anti-inflammatory, antitumor, modulate memory, neuroprotective, anti-depressive	NA	[3]
				L. loudonii	leaf				[3]
				L. speciosa	leaf				[3]
				L. villosa	leaf				[3]
264	(+)-episyring-4-O-β-D-glucopyranoside			*L. indica*	stem and leaf	NA	NA	NA	[39]

265	(2S)-α-(9′Z,12′Z)-octadecatrienoic acid monoglyceride			*L. speciosa*	fruit	NA	NA	NA	[52]
266	(2S)-α-(9′Z,12′Z,15′Z)-octadecatrienoic acid monoglyceride			*L. speciosa*	fruit	NA	NA	NA	[52]
267	(2S)-α-palmitic acid monoglyceride			*L. speciosa*	fruit	NA	NA	NA	[52]
268	(2α,3β)-urs-12-ene-2,3,28-triol			*L. speciosa*	leaf	NA	NA	NA	[17]
269	1,2-(3-hydroxy-4-methoxyphenyl)-3-oxopropyl acetate			*L. indica*	stem and leaf	NA	NA	NA	[39]
270	1,2-Propanediol,3-(1-pyrrolidinyl)-	85391-19-1	174452	*L. speciosa*	leaf	NA	NA	NA	[3]
271	1,30-Triacontanediol		543982	*L. villosa*	leaf	NA	NA	NA	[3]
272	1,3-di-O-galloyl-4,6-hexahydroxydiphenoyl-β-4C1-glucopyranose			*L. indica*	leaf	NA	NA		[38]
273	17-Pentatriacontane			*L. speciosa*	leaf	NA	NA	NA	[3]
274	2,4,6-trimethoxyphenol-1-O-β-D-(6'-O-galloyl)-glucopyranoside			*L. indica*	stem	NA	NA	NA	[10]
275	2-methoxy-5-hydroxymethyl-phenyl-1-O-(6''-galloyl)-β-D-glucopyranoside			*L. indica*	stem	NA	NA	NA	[10]
276	3,3'-O-dimethylellagic acid			*L. balansae*	stem	antimalarial	NA	NA	[35]

277	3,4,5-trimethoxyphenol 1-O-(6'-O-galloyl)-β-D-glucopyranoside			*L. balansae*	stem	NA	NA	NA	[35]
278	3,7,11,15-tetramethyl-2-hexadecen-1-ol	7541-49-3	5366244	*L. indica*	leaf	NA	NA	NA	[3]
				L. loudonii	leaf				[3]
				L. speciosa	leaf				[3]
				L. villosa	leaf				[3]
279	31-norlargerenol acetate			*L. speciosa*	leaf	NA	NA	NA	[5]
280	3-methoxyellagic acid			*L. indica*	leaf	NA	NA	NA	[38]
281	3-Methyl-5-furandione			*L. speciosa*	seed	NA	NA	NA	[19]
282	3-Methylenedihydro-2,5-furandione	2170-03-8	75110	*L. speciosa*	seed	NA	NA	NA	[19]
283	3'-O-methylellagic acid 4-O-β-D-glucopyranoside			*L. balansae*	stem	inhibit fatty acid synthase	NA	NA	[35]
284	3-O-methylgallate		54707974	*L. indica*	leaf	NA	NA	NA	[38]
285	3-O-methylprotocatechuic acid			*L. speciosa*	leaf	NA	NA	NA	[33]
286	3β-acetoxyolean-12-en-28-acid			*L. indica*	stem	NA	NA	NA	[10]
287	4-(3-hydroxy-5-(4-hydroxyphenyl)pentyl) -2-methoxyphenol			*L. indica*	stem and leaf	NA	NA	NA	[9]
288	4-(8-methoxy-1,3a,4,5a,6,7-hexahydrocyclopenta			*L. indica*	stem and leaf	NA	NA	NA	[9]
289	4-hydroxy-3-methoxyphenol 1-O-			*L. balansae*	stem	NA	NA	NA	[35]
				L. indica	stem			NA	[10]

	(6'-O-galloyl)-β-D-glucopyranoside								
290	5,5'-dibutoxy-2,2'-bifuran			*L. indica*	stem and leaf	NA	NA	NA	[9]
291	5-Methoxy-2-oxoestra-1(10),3-dien-17-yl acetate		541211	*L. speciosa*	leaf	NA	NA	NA	[3]
292	6R,9R-3-oxo-α-ionol-9-O-β-D-glucopyranoside			*L. indica*	stem	NA	NA	NA	[10]
293	9,12-Octadecadienoic acid ethyl ester	544-35-4	5282184	*L. loudonii*	leaf	anti-inflammatory, acne vulgaris, antioxidation, antiherpetic	burns, atherosclerosis, anti-inflammatory	NA	[3]
294	acacetin-7-O-6″malonylgalactoside			*L. ovalifolia*		NA	NA	NA	[22]
295	apigenin-7-O-$^4C_1$-β-D-glucoside			*L. indica*	leaf	NA	NA	NA	[38]
296	apigenin-7-*O*-acetyl-glucoside isomer			*L. ovalifolia*		NA	NA	NA	[22]
297	arjunolic acid 28-O-glucopyranoside			*L. indica*	stem	NA	NA	NA	[10]
298	astralagin			*L. indica*	leaf	NA	NA	NA	[38]
299	brevifolin	90-24-4	66654	*L. indica*	leaf	antitumor	NA	effect on human neutrophils	[38]
300	caffeoyl tartaric acid derivative			*L. ovalifolia*		NA	NA	NA	[22]
301	Catechine (2R-E)-, 5TMS derivative		13259749	*L. speciosa*	bark	NA	NA	NA	[37]

302	chlorophyll A	479-61-8	12085802	*L. indica*	leaf	antioxidant, antitumor, antibacterial, anti-inflammatory	NA	NA	[7]
303	chlorophyll B	519-62-0	11593175	*L. indica*	leaf	antioxidant, anti-inflammatory	NA	NA	[7]
304	cis-11-Eicosenamide	10436-08-5	5365374	*L. loudonii*<br>*L. speciosa*<br>*L. villosa*	leaf<br>leaf<br>leaf	NA	NA	NA	[3]<br>[3]<br>[3]
305	cis-13,16-Docasadienoic acid	17735-98-7	5312554	*L. speciosa*	seed	NA	NA	NA	[19]
306	cis-3-Tetradecene	41446-67-7	5362709	*L. speciosa*	seed	NA	NA	NA	[19]
307	cycloeucalenol acetate	10376-42-8	14282742	*L. speciosa*	leaf	NA	NA	NA	[5]
308	cytochalasin H	53760-19-3	101706637	*L. speciosa*	fruit	antitumor	antitumor	NA	[52]
309	decarboxy ellagic acid			*L. indica*	leaf	NA	NA	NA	[38]
310	dihydroactinidiolide	15356-74-8	27209	*L. indica*	leaf	NA	NA	NA	[3]
311	dihydrophaseic acid 4'-O-β-D-glucopyranoside	78914-56-4	162639198	*L. indica*	stem	NA	NA	NA	[10]
312	diisooctyl phthalate	27554-26-3	33934	*L. indica*	leaf	NA	NA	NA	[3]
313	dodecane, 4,6-dimethyl-	61141-72-8	545627	*L. loudonii*<br>*L. speciosa*<br>*L. villosa*	leaf<br>leaf<br>leaf	NA	NA	NA<br>NA	[3]<br>[3]<br>[3]
314	dodecyl acrylate	2156-97-0	75084	*L. speciosa*	seed	NA	NA	NA	[19]
315	dotriacontanol	6624-79-9	96117	*L. speciosa*	leaf	NA	NA	NA	[17]
316	dotriacotanol			*L. loudonii*	fruit	NA	NA	NA	[27]

317	Eicosanoic acid methyl ester	1120-28-1	14259	*L. loudonii*	fruit	NA	NA	NA	[27]
318	epoxycytochalasin H	80618-96-8	6440671	*L. speciosa*	fruit	NA	NA	NA	[52]
319	ethyl α-d-glucopyranoside			*L. indica*	leaf	NA	NA	NA	[3]
				L. loudonii	leaf				[3]
				L. speciosa	leaf				[3]
				L. villosa	leaf				[3]
320	ficuscarpanoside A			*L. balansae*	stem	NA	NA	NA	[18]
321	gallic acid 4-methyl ether	4319-02-2	78016	*L. indica*	stem	anti-inflammatory	NA	NA	[10]
322	glycerol β-palmitate	23470-00-0	123409	*L. indica*	leaf	antitumor	anorexia nervosa	NA	[3]
				L. loudonii	leaf				[3]
				L. speciosa	leaf				[3]
				L. villosa	leaf				[3]
323	hentriacotane			*L. loudonii*	fruit	NA	NA	NA	[27]
324	hexacosanol	506-52-5	68171	*L. loudonii*	fruit	NA	diabetes complications	NA	[27]
325	methyl palmitate	112-39-0	8181	*L. loudonii*	fruit	anti-inflammatory, antifibrotic, antitumor	anti-inflammatory, liver protection, antitumor, hypertension, anti-obesity	NA	[27]
326	lagerflorin			*L. indica*	leaf	NA	NA	NA	[2]
327	lagerindiside			*L. indica*	stem	NA	NA	NA	[10]
328	lagerine		101473393	*L. indica*	aerial part	NA	NA	NA	[50]
329	largerenol acetate			*L. speciosa*	leaf	NA	NA	NA	[5]
330	Methane, tris(methylthio)-	5418-86-0	138491	*L. indica*	leaf	NA	NA	NA	[3]
331	myrciaphenone A	26089-54-3	179470	*L. indica*	stem	NA	NA	NA	[10]
332	n-dotriacotanol			*L. speciosa*	leaf	NA	NA	NA	[53]

333	nilocitin			*L. indica*	leaf	NA	NA	NA	[38]
334	norgestrel	797-63-7	13109	*L. speciosa*	bark	NA	NA	contraception, endometrial cancer	[37]
335	octacosanol	557-61-9	68406	*L. loudonii*	fruit	hypercholesteremia, antitumor	anti-inflammatory, anti-obesity, fatigue, Parkinsonian disorders, insulin resistance	dyslipidemias, high cholesterol	[27]
336	octadecanal	638-66-4	12533	*L. loudonii*	fruit	NA	NA	NA	[27]
337	methyl stearate	112-61-8	8201	*L. loudonii*	fruit	antibacterial	neuroprotection, cerebral ischemia	NA	[27]
338	octan-2-yl 3-chlorobenzoate		12469265	*L. speciosa*	seed	NA	NA	NA	[19]
339	*p*-methoxy gallic acid methyl ester			*L. indica*	leaf	NA	NA	NA	[38]
340	quadranoside I		10416826	*L. indica*	stem	inhibit cAMP phosphodiesterase 4 isozyme	NA	NA	[10]
341	sesamin	607-80-7	72307	*L. calyculata*	stem bark	antitumor, anti-inflammatory, antioxidant, neuroprotective	atherosclerosis, anti-inflammatory, reno-protective, liver protection, neuroprotective, hyperlipidemias	sleeping disorders	[23]
342	sitosterol acetate	915-05-9	5354503	*L. speciosa*	leaf	NA	NA	NA	[5]
343	stigmast-5-en-3-ol,oleate		20831071	*L. speciosa*	leaf	NA	NA	NA	[3]
344	stigmastan-3,5-diene		525918	*L. indica*<br>*L. speciosa*	leaf<br>seed	NA	NA	NA	[3]<br>[19]

345	tellimagrandin		442690	*L. indica*	leaf	antitumor, anti-acne vulgaris	anti-inflammatory	NA	[38]
346	trans-ferulic acid actacosyl ester			*L. balansae*	stem	NA	NA	NA	[18]
347	triacotanol			*L. loudonii*	fruit	NA	NA	NA	[27]
348	tritriacotane			*L. loudonii*	fruit	NA	NA	NA	[27]
349	vomifoliol 9-O-β-D-glucopyranoside	54835-70-0	132587006	*L. indica*	stem	NA	NA	NA	[10]
350	yunnanensin A			*L. indica*	stem and leaf	NA	NA	NA	[39]
351	α-Glyceryl linoleate	67968-46-1	6436630	*L. indica*	leaf	NA	NA	NA	[3]
352	α-tocopherol	59-02-9	14985	*L. indica*<br>*L. loudonii*<br>*L. speciosa*<br>*L. villosa*	leaf<br>leaf<br>leaf, seed<br>leaf	antioxidant, anti-inflammatory, antitumor	cardiovascular, antitumor, neurodegenerative diseases, anti-inflammatory, liver protection	beta thalassemia major anemia, non-small cell lung cancer	[3]<br>[3]<br>[3]<br>[3]
353	α-tocopherolquinone	7559-04-8	24205	*L. indica*<br>*L. loudonii*<br>*L. villosa*	leaf<br>leaf<br>leaf	anti Alzheimer disease	hypercholesterolemia	NA	[3]<br>[3]<br>[3]
354	β-hydroxypropiovanillone 3-O-β-D-glucopyranoside			*L. indica*	stem	NA	NA	NA	[10]
355	β-sitosterol-3-O-β-D-(6'-O-palmityl) glucopyranoside			*L. speciosa*	leaf	NA	NA	NA	[53]
356	γ-tocopherol		45356270	*L. indica*<br>*L. loudonii*<br>*L. speciosa*	leaf<br>leaf<br>seed	NA	NA	endothelial dysfunction	[3]<br>[3]<br>[19]

				L. villosa	leaf				[3]
357	(*6S,7E,9R*)-blumenol A			*L. tomentosa*	branch	NA	NA	NA	[8]
358	3,4-dimethoxyphenol 1-O-(6'-Ogalloyl)-β-D-glucopyranoside			*L. balansae*	stem	NA	NA	NA	[35]
359	4-hydroxy-3,5-dimethoxybenzoic acid 4-O-(6'-O-galloyl)β-D-glucopyranoside			*L. balansae*	stem	NA	NA	NA	[35]
360	7-dehydrodiosgenin	85706-84-9	314392	*L. indica*<br>*L. speciosa*	leaf<br>seed	NA	NA	NA	[3]<br>[19]
361	cholesterol margarate	24365-37-5	14077841	*L. speciosa*	seed	NA	NA	NA	[19]
362	*n*-triacontanol	593-50-0	68972	*L. balansae*	stem	NA	NA	NA	[18]
363	2Z,4E,1'R,2'S,4'R,6'S-dihydrophaseic acid 4'-O-(6''-O-galloyl)-β-D-glucopyranoside			*L. balansae*	stem	NA	NA	NA	[35]
364	Vitamin E			*L. speciosa*<br>*L. villosa*	leaf<br>leaf	antioxidant, anti-inflammatory, antitumor	cardiovascular, antitumor, neurodegenerative diseases, anti-inflammatory, liver protection	nonalcoholic fatty liver disease, hyperammonemia syndrome, anal fissure, Alzheimer's disease, burn, atherosclerosis, prostate cancer, anemia	[3]<br>[3]

Note: PubChem CID numbers were obtained from https://pubchem.ncbi.nlm.nih.gov/. The *in vitro* and *in vivo* functions of the compounds were collected from the PubChem (https://pubchem.ncbi.nlm.nih.gov/), CHEMBL (https://www.ebi.ac.uk/chembl/), DrugBank (https://go.drugbank.com/), and ClinicalTrials.gov (https://clinicaltrials.gov/) databases. NA stands for "No Available", indicating that there is no record of in vitro or in vivo functions for the compound in the relevant databases.

References

1. Rabi, S.; Islam, M.N.; Islam, M.D.; Sutradhar, R.K. Bioactive Terpenoid from the Fruits of *Lagerstroemia speciosa* and Its Molecular Docking Study. *Chem. Nat. Compd.* **2022**, *58*, 485–490. https://doi.org/10.1007/s10600-022-03716-8.
2. Jeelani, S.; Khuroo, M.A. A New Pentacyclic Triterpenoid from *Lagerstroemia indica*. *Chem. Nat. Compd.* **2014**, *50*, 681–683. https://doi.org/10.1007/s10600-014-1051-4.
3. Sirikhansaeng, P.; Tanee, T.; Sudmoon, R.; Chaveerach, A. Major Phytochemical as γ -Sitosterol Disclosing and Toxicity Testing in *Lagerstroemia species*. *Evid. -Based Complement. Altern. Med.* **2017**, *2017*, 7209851. https://doi.org/10.1155/2017/7209851.
4. Chen, Y.; Li, S.-W.; Yin, F.-Z.; Yang, M.; Huan, X.-J.; Miao, Z.-H.; Wang, X.-M.; Guo, Y.-W. Lagerindicine, a New Pyrrole Alkaloid Isolated from the Flowers of *Lagerstroemia Indica* Linnaeus. *Nat. Prod. Bioprospect.* **2021**, *11*, 73–79. https://doi.org/10.1007/s13659-020-00273-x.
5. Ragasa, C.; Ngo, H.T.; Rideout, J.A. Terpenoids and Sterols from *Lagerstroemia Speciosa*. *J. Asian Nat. Prod. Res.* **2005**, *7*, 7–12. https://doi.org/10.1080/10286020310001596024.
6. Yan, F.; Huang, Y.; Wang, Y.; Li, Q.; He, X. Bioactive Sterols and Triterpenoids from the Fruits of Giant Crepe-Myrtle. *Ind. Crops Prod.* **2019**, *130*, 363–370. https://doi.org/10.1016/j.indcrop.2019.01.004.
7. Elsawi, S.A.; Aly, H.F.; Elbatanony, M.M.; Maamoun, A.A.; Mowawad, D.M. Phytochemical Evaluation of *Lagerstroemia indica* (L.) Pers Leaves as Anti-Alzheimer's. *J. Mater. Environ. Sci.* **2018**, *9*, 2575–2586.
8. Hui, D.; Zhang, R.; Xu, L.; Jie, J.; Zhou, C.; Yu, Z. Constituents of Three Species of *Lagerstroemia*. *Biochem. Syst. Ecol.* **2005**, *33*, 639–642. https://doi.org/10.1016/j.bse.2004.10.012.
9. Zhang, D. Studies on the Chemical Constituents and Bioactivities in Stems and Leaves of *Lagerstroemia indica* L. Master's Dissertation, Huaqiao University, Quanzhou, China, 2016.
10. Woo, K.W.; Cha, J.M.; Choi, S.U.; Lee, K.R. A New Triterpene Glycoside from the Stems of *Lagerstroemia indica*. *Arch. Pharmacal Res.* **2016**, *39*, 631–635. https://doi.org/10.1007/s12272-016-0746-4.
11. Chaudhuri, P.K. A Labdane Diterpenoid Sterol from *Lagerstroemia Lancasteri*. *Phytochemistry* **1987**, *26*, 3361–3362. https://doi.org/10.1016/S0031-9422(00)82511-X.
12. Choi, J.; Ku, P.-T.; Cho, K.-S.; Huh, M.-K. Comparison of Chemicals in *Lagerstroemia spezzciosa* (L.) Pers. at Growing Stage Levels by GC-MS. *Korean J. Crop Sci.* **2010**, *55*, 200–206.
13. Okada, Y.; Omae, A.; Okuyama, T. A New Triterpenoid Isolated from *Lagerstronemia speciosa* (L.) Pers. *Chem. Pharm. Bull.* **2003**, *34*, 452–454. https://doi.org/10.1248/cpb.51.452.
14. Bani, T.; Prabir, K.; Chaudhuri, A.K.; Mallik Lagerenyl Acetate and Lagerenol Two Tetracyclic Triterpenoids with the Cycloartane Skeleton from *Lagerstroemia lancasteri*. *Phytochemistry* **1983**, *22*, 2559–2562. https://doi.org/10.1016/0031-9422(83)80164-2.
15. Qi, S.; Wu, D.; Ma, Y.; Wu, S.; Mei, W.; Luo, X. Studies on Chemical Constituents of *Lagerstroemia guilinensis*. *Chin. Tradit. Herb. Drugs* **2002**, *33*, 17-18. https://doi.org/10.3321/j.issn:0253-2670.2002.10.006.
16. Osawa, K.; Ueda, J.; Takahashi, M. The Components of the Plants of *Lagerstroemia* Genus. II. Studies on the Components of the Leaves of *Lagerstroemia Speciosa* (L.) Pers., *L. Subcostata* Koehne., *L. Indica* Linn., and L. Fauriei Koehne. *Yakugaku zasshi.* **1974**, *94*, 271–273. https://doi.org/10.1248/yakushi1947.94.2_271.
17. Zhan, Q.; Wang, Y.; Li, X.; Chen, W.; Sun, L. Studies on the Chemical Constituents of Petroleum Ether Extract of *Lagerstroemia Speciosa* (Linn.) Pers Leaves. *Lishizhen Med. Mater. Med. Res.* **2009**, *20*, 2125–2127. https://doi.org/10.3969/j.issn.1008-0805.2009.09.008.
18. Zhou, Y.; Wang, B.; Zhang, Q.; Chen, H. Chemical Constituents of *Lagerstroemia balansae* Koehne. *Chin. Pharm. J.* **2012**, *45*, 169–171.
19. Raju, L.; Lipin, R.; Eswaran, R. Identification, ADMET Evaluation and Molecular Docking Analysis of Phytosterols from Banaba (*Lagerstroemia speciosa* (L.)Pers) Seed Extract against Breast Cancer. *Silico Pharmacol.* **2021**, *9*, 43. https://doi.org/10.1007/s40203-021-00104-y.

20. Hou, W.; Li, Y.; Zhang, Q.; Wei, X.; Peng, A.; Chen, L.; Wei, Y. Triterpene Acids Isolated from *Lagerstroemia Speciosa* Leaves as α-Glucosidase Inhibitors. *Phytother. Res.* **2009**, *23*, 614–618. https://doi.org/10.1002/ptr.2661.
21. Huang, G.-H.; Zhan, Q.; Li, J.-L.; Chen, C.; Huang, D.-D.; Chen, W.-S.; Sun, L.-N. Chemical Constituents from Leaves of *Lagerstroemia Speciosa* L. *Biochem. Syst. Ecol.* **2013**, *51*, 109–112. https://doi.org/10.1016/j.bse.2013.08.029.
22. Kim, M.O.; Su, U.L.; Yuk, H.J.; Jang, H.J.; Ryu, H.W. Metabolomics Approach to Identify the Active Substances Influencing the Antidiabetic Activity of Lagerstroemia Species. *J. Funct. Foods* **2019**, *64*, 103684.
23. Lou, X.; Zhang, R.; Zhao, Y.; Dou, H.; Zou, C. Chemical Studies on the Leaves of *Lagerstroemia Reginae* Roxb. *Nat. Prod. Res. Dev.* **2006**, *18*, 962–963. https://doi.org/10.16333/j.1001-6880.2006.06.018.
24. Ashnagar, A.; Ghanad, A.R.; Motakefpour, M. Isolation and Identification of Major Chemical Components Found in the Leaves of *Lagerstroemia indica* Plant Grown in the City of Tehran, Iran. *Int. J. ChemTech Res.* **2013**, *5*, 478–481.
25. Riyanti, S.; Dewi, P.S.; Windyaswari, A.S.; Azizah, S.A.N. Alpha-Glucosidase Inhibitory Activities of Bungur (*Lagerstroemia loudonii* Teijsm. & Binn.) Leaves and Fruits. *IOP Conf. Ser. Earth Environ. Sci.* **2020**, *462*, 012042. https://doi.org/10.1088/1755-1315/462/1/012042.
26. Barik, B.R.; Kundu, A.B. Lageflorin, a Pentacyclic Triterpene from *Lagerstroemia Parviflora*. *Phytochemistry* **1988**, *27*, 3679–3680, https://doi.org/10.1016/0031-9422(88)80796-9.
27. Boonphong, S. Waxes and Triterpene Acids from *Lagerstroemia loudonii* Fruit. *NU. Int. J. Sci.* **2013**, *10*, 33–43.
28. Hussain, F.; Ganguly, A.; Hossain, M.S.; Rahman, S.A. Analgesic and Anti-Diarrhoeal Activities of *Lagerstroemia speciosa* Roots in Experimental Animal Model. *Dhaka Univ. J. Pharm. Sci.* **2014**, *13*, 57–62. https://doi.org/10.3329/dujps.v13i1.21860.
29. Zhan, Q.; Wang, Y.; Li, X.; Yang, Y.; Chen, W.; Sun, L. Studies on the Chemical Constituents of Ethyl Acetate Extract from *Lagerstroemia Speciosa* (Linn.) Pers Leaves. *Lishizhen Med. Mater. Med. Res.* **2009**, *20*, 1841–1842. https://doi.org/10.3969/j.issn.1008-0805.2009.08.002.
30. Hussain, S.F.; Miana, G.A.; Saifur, R. 3,4,3′-Tri-O-Methylellagic Acid from *Lagerstroemia Indica*. *Phytochemistry* **1972**, *11*, 2890–2891. https://doi.org/10.1016/s0031-9422(00)86540-1.
31. Saleh, N.A.M. Anthocyanins of *Lagerstroemia Indica* Flowers. *Phytochemistry* **1973**, *12*, 2304. https://doi.org/10.1016/0031-9422(73)85145-3.
32. Kolakul, P.; Sripanidkulchai, B. Phytochemicals and Anti-Aging Potentials of the Extracts from *Lagerstroemia speciosa* and Lagerstroemia *Floribunda*. *Ind. Crops Prod.* **2017**, *109*, 707–716. https://doi.org/10.1016/j.indcrop.2017.09.026.
33. Bai, N.; He, K.; Roller, M.; Zheng, B.; Chen, X.; Shao, Z.; Peng, T.; Zheng, Q. Active Compounds from *Lagerstroemia speciosa*, Insulin-like Glucose Uptake-Stimulatory/Inhibitory and Adipocyte Differentiation-Inhibitory Activities in 3T3-L1 Cells. *J. Agric. Food Chem.* **2008**, *56*, 11668–11674. https://doi.org/10.1021/jf802152z.
34. Osawa, K.; Ueda, J.; Takahashi, M. The Components of the Plants of *Lagerstroemia* Genus. II. Studies on the Components of the Leaves of *Lagerstroemia Speciosa* (L.) Pers., *L. Subcostata* Koehne., *L. Indica* Linn., and L. Fauriei Koehne. *Yakugaku zasshi.* **1974**, *94*, 271–273. https://doi.org/10.1248/yakushi1947.94.2_271.
35. Zhou, Y.; Chen, H.; Wang, B.; Liang, H.; Zhao, Y.; Zhang, Q. Sesquiterpenoid and Phenolic Glucoside Gallates from *Lagerstroemia balansae*. *Planta Med.* **2011**, *77*, 1944–1946. https://doi.org/10.1055/s-0031-1280093.
36. Hosoyama, H.; Sugimoto, A.; Suzuki, Y.; Sakane, I.; Kakuda, T. Isolation and Quantitative Analysis of the Alpha-Amylase Inhibitor in *Lagerstroemia speciosa* (L.) Pers.(Banaba). *Yakugaku Zasshi J. Pharm. Soc. Jpn.* **2003**, *123*, 599–605.
37. Pal, L.C.; Kumar, A.; Pande, V.; Ch.V.; Rao, R. Hepatoprotective Effect of Bioactive Fraction of *Lagerstroemia speciosa* (L.) Pers. Bark Against Monosodium Glutamate-Induced Liver Toxicity. *Pharmacogn. J.* **2020**, *12*, 1630–1640. https://doi.org/10.5530/pj.2020.12.223.
38. Labib, R.M.; Ayoub, N.A.; Singab, A.B.; Al-Azizi, M.M.; Sleem, A. Chemical Constituents and Pharmacological Studies of *Lagerstroemia indica*. *Phytopharmacology* **2013**, *4*, 373–389.

39. Zhang, D. Chemical Constituents in Stem-Leaves of *Lagerstroemia Indica*. *Chinese Traditional and Herbal Drugs* **2015**, *46*, 2209–2211. https://doi.org/10.7501/j.issn.0253-2670.2015.15.005.
40. Song, J.H.; Park, K.S.; Kwon, D.H.; Choi, H.J. Anti–Human Rhinovirus 2 Activity and Mode of Action of Quercetin-7-Glucoside from *Lagerstroemia speciosa*. *J. Med. Food* **2013**, *16*, 274–279. https://doi.org/10.1089/jmf.2012.2290.
41. Xu, Y.-M.; SAKAI, T.; TANAKA, T.; NONAKA, G.; NISHIOKA, I. Tannins and Related Compounds. CVI. Preparation of Aminoalditol Derivatives of Hydrolyzable Tannins Having α-and β-Glucopyranose Cores, and Its Application to the Structure Elucidation of New Tannins, Reginins A and B and Flosin A, Isolated from *Lagerstroemia Flos-Reginae* RETZ. *Chem. Pharm. Bull.* **1991**, *39*, 639–646. https://doi.org/10.1248/cpb.39.639.
42. Xu, Y.-M.; Tanaka, T.; Nonaka, G.; Nishioka, I. Tannins and Related Compounds. CVII. Structure Elucidation of Three New Monomeric and Dimeric Ellagitannins, Flosin B and Reginins C and D, Isolated from *Lagerstroemia Flos-Reginae* Retz. *Chem. Pharm. Bull.* **1991**, *39*, 647–650. https://doi.org/doi:10.1248/cpb.39.647.
43. Hayashi, T.; Maruyama, H.; Kasai, R.; Hattori, K.; Takasuga, S.; Hazeki, O.; Yamasaki, K.; Tanaka, T. Ellagitannins from Lagerstroemia Speciosa as Activators of Glucose Transport in Fat Cells. *Planta Medica* **2002**, *68*, 173–175. https://doi.org/10.1055/s-2002-20251.
44. Tanaka, T.; Tong, H.H.; Xu, Y.M.; Ishimaru, K.; Nonaka, G.I.; Nishioka, I. Tannins and Related Compounds. CXVII. Isolation and Characterization of Three New Ellagitannins, Lagerstannins A, B and C, Having a Gluconic Acid Core, from *Lagerstroemia Speciosa* (L.) PERS. *Chem. Pharm. Bull.* **1992**, *40*, 2975–2980. https://doi.org/10.1248/cpb.40.2975.
45. Liu, X.; Kim, J.; Li, Y.; Li, J.; Liu, F.; Chen, X. Tannic Acid Stimulates Glucose Transport and Inhibits Adipocyte Differentiation in 3T3-L1 Cells. *J. Nutr.* **2005**, *135*, 165–171.
46. Koshio, K.; Murai, Y.; Sanada, A.; Taketomi, T.; Yamazaki, M.; Kim TaeSoon; Boo HeeOck; Obuchi, M.; Iwashina, T. Positive Relationship between Anthocyanin and Corosolic Acid Contents in Leaves of *Lagerstroemia speciosa* Pars. *Trop. Agric. Dev.* **2012**, *56*, 49–52.
47. Zhang, J.; Wang, L.; Gao, J.; Shu, Q.; Li, C.; Yao, J.; Hao, Q.; Zhang, J. Determination of Anthocyanins and Exploration of Relationship between Their Composition and Petal Coloration in Crape Myrtle (*Lagerstroemia* Hybrid). *J. Integr. Plant Biol.* **2008**, *50*, 581–588. https://doi.org/10.1111/j.1744-7909.2008.00649.x.
48. Klein, G.; Kim, J.; Himmeldirk, K.; Cao, Y.; Chen, X. Antidiabetes and Anti-Obesity Activity of Lagerstroemia Speciosa. *Evid. -Based Complement. Altern. Med.* **2007**, *4*, 401–407. https://doi.org/10.1093/ecam/nem013.
49. Fuji, K.; Yamada, T.; Fujita, E.; Murata, H. Lythraceous Alkaloids. X. Alkaloids of *Lagerstroemia Subcostata* and L. Favriei: A Contribution to the Chemotaxonomy. *Chem. Pharm. Bull.* **1978**, *26*, 2515–2521. https://doi.org/10.1248/cpb.26.2515.
50. Kim, H.J.; Lee, I.S.; Youn, U.; Chen, Q.C.; Ngoc, T.M.; Ha, D.T.; Liu, H.; Min, B.S.; Lee, J.Y.; Seong, R.S. Biphenylquinolizidine Alkaloids from *Lagerstroemia Indica*. *J. Nat. Prod.* **2009**, *72*, 749–752. https://doi.org/10.1021/np800619g.
51. Watanabe, K.; Kubota, T.; Shinzato, T.; Ito, J.; Mikami, Y.; Kobayashi, J. Sarusubine A, a New Dimeric Lythraceae Alkaloid from *Lagerstroemia Subcostata*. *Tetrahedron Lett.* **2007**, *48*, 7502–7504. https://doi.org/10.1016/j.tetlet.2007.08.059.
52. Yan, F. Study on the Bioactive Constituents of the Fruits of *Lagerstroemia speciosa* (L.) Pers. Master's Dissertation, Guangdong Pharmaceutical University, Guangdong, China, 2020.
53. Zhan, Q. Study on the Hypoglycemic Constituents from Leaves of *Lagerstroemia speciosa*. Master's Dissertation, Second Military Medical University, Shanghai, China, 2008.
54. Zong, W.; Xia, W. Physicochemical Properties of Banaba Seed Oil and Its Fatty Acid Composition Determined by GC/MS. *China Oils Fats* **2004**, *29*, 65–67. https://doi.org/10.3321/j.issn:1003-7969.2004.10.018.
55. Jehan, C.M.; Daulatabad, D.; Mirajkar, A.M. A Keto Fatty Acid from *Lagerstroemia speciosa* Seed Oil. *Phytochemistry* **1990**, *29*, 2323–2324. https://doi.org/10.1016/0031-9422(90)83061-5.
56. Basu, S.; Kundu, P.; Sinhababu, A. Characterization of Fatty Acid and Sterol Composition of Seed Lipid of *Lagerstroemia speciosa* Pers. *Res. Chem. Intermed.* **2015**, *41*, 6511–6522. https://doi.org/10.1007/s11164-014-1757-8.

57. Zong, W.; Zhao, G. Analysis of the Protein and Amino Acid Contents in the Seed of Lagerstroemia Speciosa. *Mod. Food Sci. Technol.* **2006**, *22*, 227–228. https://doi.org/10.3969/j.issn.1673-9078.2006.04.076.
58. Muangsin, N.; Wisetsakdakorn, W.; Chaichit, N.; Sihanonth, P.; Petsom, A.; Sangvanich, P. Austrocortinin: Crystal Structure of a Natural Anthraquinone Pigment from Fungi. *Dyes Pigm.* **2008**, *77*, 653–656. https://doi.org/10.1016/j.dyepig.2007.09.004.
